# Shared decision making in pharmacotherapy decisions, perceived by patients with bipolar disorder

**DOI:** 10.1186/s40345-018-0129-5

**Published:** 2018-10-04

**Authors:** Doris Verwijmeren, Koen P. Grootens

**Affiliations:** 1 Reinier van Arkel Mental Health Institute, Postbus 70058, 5201 DZ ’s-Hertogenbosch, The Netherlands; 2 Department of Psychiatry, Radboud University Medical Centre, Nijmegen, The Netherlands

**Keywords:** Communication, Bipolar disorders, Pharmacotherapy

## Abstract

**Background:**

Shared decision making has been promoted as standard care, but there has been debate on the possible types. On the one hand, there is a more ‘instrumental’/objective approach focused on the exchange of information, but an ‘interpersonal’/subjective patient involvement has been suggested as well. In this study we aim to investigate this further by assessing both actual and perceived patient involvement in medical decisions.

**Methods:**

Eighty-one consultations between patients with bipolar disorder and their clinicians were observed and scored using the OPTION scale. Afterwards, the patients’ experienced involvement was explored with the SDM-Q-9. Furthermore, several patient characteristics were gathered. Correlations between the scores were examined.

**Results:**

The clinicians scored on average 34.6 points on the OPTION scale. In contrast, patients scored on average 77.5 points on the SDM-Q-9, suggesting that patients felt more involved in the consultation than was observable.

**Conclusion:**

Our patients with bipolar disorder feel involved in pharmacotherapy decisions, but this is not scored in objective observations. Our data suggest that there are implicit, interpersonal aspects of patient involvement in shared decision making, a concept that deserves further attention and conceptualisation.

## Background

### Shared decision making

In recent decades, patient participation in medical decisions has increased enormously and has become the new standard (de Cuevas et al. 2011; Hamann et al. 2011; Zijlstra and Goossensen 2007). This patient-centred care has been promoted by an increased emphasis on clinical interaction with respect to patients’ values, preferences and experiences (Elwyn et al. 2014). To explore these values and preferences, the model of shared decision making (SDM) has gradually evolved. In SDM, patient and physician discuss the pros and cons of all treatment options using the best available evidence for the individual situation. In addition, the clinician is encouraged to examine the patient’s preferences and fears and his or her preferred level of participation in the decision making (Elwyn et al. 2014; Goossensen et al. 2007; Morant et al. 2016). The most important aspect is that at the end of the process a decision is made that matches the patient’s values, is informed based on evidence, and has the full agreement of both doctor and patient (Simmons et al. 2010).

### Importance of SDM

The importance of SDM is widely recognised. The benefits, such as improved self-management (Goossensen et al. 2007), patient satisfaction (Ilgen et al. 2009) and treatment adherence (Simmons et al. 2010; Hamann et al. 2003; McCabe et al. 2013; Stacey et al. 2008; Sylvia et al. 2013) have already been described in several studies throughout the medical field. Some studies even report an improvement in clinical outcomes due to a higher level of SDM (Hamann et al. 2003; Os et al. 2005; Rathert et al. 2013). In psychiatry, a similar shift towards interest in SDM has taken place as in general medicine. Os et al. (2005) showed an improvement in patient outcomes in depression where the antidepressant treatment was provided by a doctor with good communicative skills (Os et al. 2005). Nevertheless, compared to the large number of studies assessing patient participation in decision making in somatic medicine, SDM in the mental health field is a relatively understudied topic (Morant et al. 2016; Hamann et al. 2003).

### SDM in patients with bipolar disorder

Bipolar patients are an outstanding group to involve in SDM. Patients with bipolar disorder have a great responsibility in self-managing their disease to prevent a relapse or recurrence (Fisher et al. 2016). The intermittent nature of the disorder makes it convenient to apply SDM in the symptom-free intervals. There are a variety of options for maintenance treatment that need further explanation and consideration, each with more or less similar efficacy but different side effects and monitoring aspects (Kupka et al. 2015).

Finally, treatment adherence remains a major challenge due to the significant side effects of the drugs described, such as lithium-induced renal failure. This makes a balanced consideration of the choice of treatment especially relevant (Fisher et al. 2016; Sajatovic et al. 2005). Nevertheless, a recent systematic review showed only 13 studies on SDM in bipolar patients, with only two bipolar-only samples and eleven mixed psychiatric patient samples with a minority of bipolar patients (Fisher et al. 2016).

In the studies in that review, bipolar patients preferred making decisions together with their clinician or making the decision alone. Less than a third of patients reported preferring a passive decision style. Most patients wanted to rely on the professional advice of their clinician and preferred the clinician to make the final treatment decisions (Fisher et al. 2016).

### Differences between ‘being’ and ‘feeling’ involved?

Research and policy on SDM so far seem to focus predominantly on the objective part of SDM, such as the exchange of information or treatment options. However, a more subjective approach has been suggested as well, concerning a more patient-oriented style and subjective phenomena that play a part in the consult. For instance, Fisher et al. (2016) separates the patient involvement in ‘instrumental’ and ‘interpersonal’. Also Goossensen et al. (2007) conducted a study for comparing doctor skills (measured by the OPTION scale) versus patient satisfaction and concluded that although the skills of SDM from the doctor did not always score that high, the patient satisfaction did. Entwistle and Watt (2006) suggests several aspects of involvement in decision-making., such as the patient’s views and feelings about their role and contributions in relation to decision-making. In addition, the patient’s views and feelings about their relationship with their clinician can be taken into account. Patients themselves also talk in terms of feeling involved, not just being. The authors notice that less attention has been paid to these aspects (Entwistle and Watt 2006).

Most ideally, this topic should be addressed in a qualitative study. Because there are no validated quantitative tests aligning this topic, we aim to look for directions pointing out a possible difference between this ‘being’ and ‘feeling’ involved by assessing the patient’s perspective about SDM with the SDM-Q-9.

### Study aims and hypotheses

In this study, we aimed to research the preferences of patients with bipolar disorder in the decision-making process regarding pharmacotherapeutic options. The study was designed to explore possible differences between the subjective and observable aspects of SDM. By focusing on this particular patient group and only on pharmacotherapeutic decisions, we attempt to obtain a clear and complete image of SDM. We hypothesised that patients with bipolar disorder wanted to be involved in the decision-making process, as has already been described in other studies with different patient samples in both psychiatric and somatic medicine (Blumenthal-Barby et al. 2015; Cuevas et al. 2014; Zheng et al. 2006).

In addition, we expected to find indications for a distinction between implicit, experienced and explicit, observed patient involvement. We explicit use the term indications, because we realise that the SDM-Q-9 is a scale to measure the patient’s perspective more than pure implicit communication.

## Methods

### Participants

Consultations between psychiatrists, residents, nurse practitioners and their patients were observed by the first author during real-time observation, after informed consent. No audio-or video-recordings were made. Most clinicians were partly instructed about the topic, by telling them the communication would be observed, but not revealing our hypotheses. The consultations took place in two specialised outpatient clinics for bipolar disorder in ’s-Hertogenbosch and Nijmegen in the Netherlands. The following inclusion criteria were used: age over 18 years, a known DSM-IV diagnosis of bipolar disorder I, II or ‘not otherwise specified’ (NOS) diagnosed by a psychiatrist, and a consultation which was expected to involve a pharmacotherapy decision. We specifically did not place any limitations related to current mood symptoms to create a representative image of a consultation with a patient with bipolar disorder. Patients were asked to answer a questionnaire afterwards. Participants provided both written and verbal informed consent prior to the consultation. Patients were included in the study during 8 weeks. The study was approved by the local ethics committee.

Of the 91 patients who were asked to join the research, only eight refused informed consent. Two patients did not have a bipolar disorder and were subsequently excluded from the study. Three patients did not return the questionnaire and were treated as lost to follow-up. Forty-two patients did not complete the full questionnaire. As shown in Table 1, most of the 81 patients had a DSM-IV bipolar I disorder and more than half had been admitted to hospital at least once in their lifetime.

**Table 1:** Patient characteristics

Characteristics	Sample, N = 81		
	*N*	%	*SD*
Gender			
Female	52	64.2	
Male	29	35.9	
Age (mean in years)	52.0		13.6
Ethnic origin			
Dutch	78	96.3	
American	1	1.2	
Asian	1	1.2	
African	1	1.2	
Diagnosis			
Bipolar I	50	61.7	
Bipolar II	23	28.4	
Bipolar NOS	8	9.9	
Clinician			
Psychiatrist	61	75.3	
Trainee	5	6.2	
Nurse practitioner	15	18.5	
Time of consultation (mean in minutes)	40.1		15.2
Total duration of treatment (years)			
< 1	34	42.0	
1–5	33	40.7	
5–10	12	14.8	
> 10	2	2.5	
Number of clinical admissions			
0	13	18.8	
1–3	49	71.1	
4–5	5	6.2	
> 5	2	2.4	

### Instruments

Explicit aspects of SDM were observed and scored using OPTION (Observing Patient Involvement in Treatment Choices), an instrument that measures (on a scale from 0 to 100) the degree to which clinicians involve patients in decision making (Elwyn et al. 2005a, b). The instrument consists of 12 items to observe verbal behaviour displayed by the clinician. The instrument can be applied using audio- or video-recording, or live rating (Elwyn et al. 2005a).

After the consultation, we asked the participant to fill in a questionnaire exploring the preceding process of SDM from the patient’s perspective (the implicit SDM), using the nine-item Shared Decision-Making Questionnaire (SDM-Q-9). The SDM-Q-9 is developed to measure the process of SDM by self-assessment of the patient. It consists of 9 items, each addressing a step of the SDM process (Kriston et al. 2010). In addition, the Decision Self-Efficacy Scale was used to measure the patient’s self-confidence or belief in their abilities in decision making, including SDM. Third, the WHO Disability Assessment Schedule 2.0 (WHODAS 2.0) (WHO 2017) was scored to obtain a clear image of the severity of the disease in the last 3 months. The WHODAS 2.0 is developed by the WHO organization to measure disability levels on six domains of functioning: cognition, mobility, self-care, getting along, life activities, participation (WHO 2017). The scale is available in a 36-item scale and a 12-item scale. In this research, the 12-item scale is used.

Finally, the patients were asked to answer eight self-designed multiple choice questions about the communication with their physician and their preferences regarding the communication (see Table 3). This questionnaire was designed to evaluate possible bias in our design and to further underpin our results.

### Data analysis

We used descriptives and a Pearson correlation to test the correlation between OPTION and SDM-Q-9. For statistical testing of the answers to the multiple choice questions, crosstabs with Chi square were used. Furthermore, we used a Pearson correlation and two-way ANOVA with Least Significant Difference for statistical testing of the influence of the different variables on the OPTION and SDM-Q-9 scales. A significance level of 0.05 was used. Data were analysed with SPSS, Version 22.

## Results

### Quality of SDM scored by observer

The mean OPTION score of all clinicians over the remaining 78 consultations was 34.6 (*SD* = 16.4). The mean scores per clinician ranged from 0.0 to 64.6. We explored the associations between the OPTION scale and the following variables: different clinicians, patient sex, patient age, diagnosis, self-confidence for making a decision, severity of bipolar symptoms (WHODAS 2.0 and number of clinical admissions), duration of total treatment by the same clinician and duration of the consultation.

Only duration of consultation was found to have a statistically significant relationship with the observed OPTION scale. The correlation was: the longer the consultation, the higher the OPTION score (see Table 2).

**Table 2:** Correlation between different patient- and consultation-specific variables and the OPTION scale (explicit shared decision making) and the SDM-Q-9 (implicit shared decision making)

Variable	OPTION		SDM-Q-9	
	R**	*p*	*R*	*p*
Duration of consultation	0.39	0.00*	0.14	0.25
Number of clinical admissions	− 0.20	0.11	− 0.25	0.046*
Decision Self-Efficacy Scale	− 0.07	0.59	0.52	0.00*
Duration of total treatment	− 0.15	0.19	− 0.09	0.45
OPTION	–	–	0.08	0.51

Statistically significant results are in italics

* Correlation is significant at the 0.05 level

** R = Pearson correlation

### Quality of SDM scored by the patient

The mean SDM-Q-9 score for the patients was 77.5 (*SD* 23.5, range 6.7–100). We also explored the relationships between the patient characteristics and the SDM-Q-9. We found a positive relationship between the number of clinical admissions and the SDM-Q-9 (*p* = 0.046), suggesting that the severity of the disease influences the patient’s feeling of being involved in the decision-making process.

We also found a significant relationship between the Decision Self-Efficacy Scale and the SDM-Q-9 (Table 2). In contrast to our hypothesis, we found no correlation between the SDM-Q-9 and the total duration of treatment.

In line with our hypothesis, there was no correlation between the OPTION score and SDM-Q-9 (*r* = 0.08 and statistically not significant). Despite the fact that the two scales cannot be compared, we found a higher mean on the SDM-Q-9 of 42.9, indicating that, in the patients’ opinion or feeling, the use of SDM is higher than the researcher observed.

### Needs of patients with bipolar disorder

The results of the multiple choice questions are presented in Table 3. As shown in the table, the majority of patients find it very important that both doctor and patient agree on the final decision. Furthermore, it is clear that most patients prefer that both patient and doctor are involved in the decision. If the patient were (theoretically) forced to choose between the two, half of the patients said they would make the decision themselves and the other half would let the clinician decide. Only ‘age of the patient’ was found to be significantly related to this aspect. It appeared that the older the patient is, the more he or she prefers a final decision by the doctor (*p* = 0.021).

**Table 3:** Patients’ answers to multiple choice questions in the qualitative interview

Question	Answer	
	*N*	%
1. How did you experience the communication concerning the decision making by your doctor in comparison with previous consultations?		
A. In this conversation I was more involved in the decision-making process than usually	12	16.2
B. In this conversation I was less involved in the decision-making process than usually	1	1.4
C. The doctor behaved in the same way as usually	61	82.4
2. Did you ever use decision aids to make a decision about your treatment? If yes: which decision aids did you use?^a^		
A. Yes	18	25.0
B. No	54	75.0
3. Do you think you would benefit from different tools when making a decision about your treatment? E.g. on the internet, a flyer or an app. If yes, go to question 4. If no, go to question 6		
A. Yes	26	38.2
B. No	42	61.8
4. In the previous question you indicated your need for decision aids. Which tools would you prefer? Multiple answers possible		
A. The pros and cons of each treatment	24	77.4
B. The effects and side effects of each treatment	23	74.2
C. Questions about your preferences which guide you to make a decision about your treatment	7	22.6
D. A proposal of critical questions to ask your doctor for more information about the different treatment options	11	35.5
5. What kind of format would you prefer to present the above chosen aids?		
A. Folder	18	54.5
B. Website	12	36.4
C. An app for your smartphone	3	9.1
6. How important do you think it is that you and your doctor agree on the decision made? (on a scale from 0 to 5)		
0: Not important at all	0	0.0
1: Mostly not important	1	1.9
2: Partly not important	0	0.0
3: Partly important	5	9.6
4: Mostly important	13	25.0
5: Very important	33	63.5
7. If you and your doctor have to make a decision, who makes the decision in the end?		
A. Your doctor	7	13.5
B. You	11	21.2
C. Both of you	34	65.4
8. If you and your doctor disagree about a decision, whose opinion do you eventually follow?		
A. The doctor’s opinion	25	52.1
B. Your own opinion	23	47.9

^a^Patients who did use decision aids before used mostly internet, books and advice from their social environment/support group

### Multiple analysis

In the two-way ANOVA of all the independent factors mentioned in “Quality of SDM scored by the patient” section we found only duration of consultation to be of influence on the OPTION scale (*F* = 5.51 and *p* = 0.01) (Table 4). After adjustment for multiple comparisons by Least Significant Difference we found comparison of the shortest duration of consultation (0–25 min) with the longest duration of consultation (51–75 min) to be still significant (*p* = 0.003). Comparison of the 26–50 min group with the longest time was also significant, with a p-value of 0.017.

**Table 4:** ANOVA summary table for OPTION scale

Source	*df*	MS	F	*p*	Effect size
Gender	1	2.293	0.009	0.924	0.000
Diagnosis	2	91.247	0.365	0.697	0.026
Age	3	262.605	1.052	0.386	0.105
Clinician	2	206.761	0.828	0.448	0.058
Duration of total treatment	3	263.793	1.056	0.384	0.105
SDM-Q-9	1	5.781	0.023	0.880	0.001
WHODAS 2.0	1	350.003	1.402	0.247	0.049
Duration of consultation	2	1374.875	5.506	*0.010* *	0.290
Decision Self-Efficacy scale	2	489.703	1.961	0.160	0.127
Error	27	249.719			
Total	47				

Statistically significant results are in italics, R^2^ = 0.508 (adjusted R^2^ = 0.161)

*MS*  mean squares, effect size = partial η^2^

* Statistically significant at the 0.05 level

In the two-way ANOVA of the SDM-Q-9 the only independent factor of influence on the SDM-Q-9 was the Decision Self-Efficacy Scale (*F* = 3.82 and *p* = 0.35) (Table 5). After adjustment for multiple comparisons by Least Significant Difference we found a significant difference between the lowest score on this scale (0–50) and the highest score (76–100) with a p-value of 0.02.

**Table 5:** ANOVA summary table for SDM-Q-9

Source	*df*	MS	F	*p*	Effect size
Gender	1	521.908	1.051	0.314	0.037
Diagnosis	2	674.905	1.360	0.274	0.091
Age	3	931.708	1.877	0.157	0.173
Clinician	2	167.655	0.338	0.716	0.024
Duration of total treatment	3	333.630	0.672	0.577	0.069
OPTION	1	16.581	0.033	0.856	0.001
WHODAS 2.0	1	830.694	1.673	0.207	0.058
Duration of consultation	2	734.410	1.479	0.246	0.099
Decision Self-Efficacy scale	2	1897.546	3.822	*0.035* *	0.221
Error	27	496.420			
Total	47				

Statistically significant results are in italics, R^2^ = 0.549 (adjusted R^2^ = 0.232)

*MS* mean squares, effect size = partial η^2^

* Statistically significant at the 0.05 level

## Discussion

The study presented here was designed to research the opinion of patients with bipolar disorder about their desired and experienced (implicit) involvement, combined with the observed (explicit) involvement in the shared decision-making process.

The clinicians in our sample scored relatively high on the OPTION scale in comparison with other psychiatrists (Goossensen et al. 2007; McCabe et al. 2013) and with paediatricians (Brinkman et al. 2011), general practitioners (Hamann et al. 2003) and oncologists (Arend 2006). Although direct comparisons between the OPTION scale and SDM-Q-9 are not possible, we state that the implicit SDM (experienced involvement) is higher than the observed/explicit SDM. There is no correlation found between the SDM-Q-9 and the OPTION scores.

Although the SDM-Q-9 is not developed for the interpersonal aspect of SDM, the fact that the patient rated the consultation much higher than the observer, supports our hypothesis that SDM involves both an explicit, observable aspect (information, patient values, consideration of all options) and an implicit, interpersonal aspect that needs further attention, as suggested in the literature mentioned in “Differences between ‘being’ and ‘feeling’ involved?” section.

The difference between the implicit and explicit SDM might be explained by the importance of the clinicians’ non-verbal behaviour in contrast to the verbal exchange and explanations. The inner feeling of the patient, and possibly the confidence and the relationship with the clinician may be much more important.

In addition, it is possible that patients have a blind spot for possible improvement in their treatment. Patients are content with this communication because they don’t realize that improvement is possible. In addition, from a methodically point of view, in general patients tend to be positive about their clinicians in questionnaire research (Edwards et al. 2004).

In exploring the different variables influencing both the OPTION score and SDM-Q-9, only time of consultation and self-efficacy and number of clinical admissions were correlated with OPTION respectively SDM-Q-9. Interestingly, experienced involvement was not correlated with the duration of the consultation, another clue that involvement is not only about the transmission of information. In contrast to our hypothesis, the duration of the total treatment history had no influence on the implicit and the explicit SDM. This may be partly because there is no normal distribution in our sample, as around 80% of the patients had a treatment duration of < 5 years. Unfortunately, there are no studies focusing on the duration of treatment as a possible influence on SDM.

Cuevas et al. (2014) found that having low efficacy predicted for the patient’s experience that the doctor used a passive decision-making style, and little use of SDM. This is in accordance with our findings that a lower score on the Decision Self-Efficacy Scale led to a lower score on the SDM-Q-9. This is not surprising, as it described in literature that there is increased use of a more passive decision-making style by the doctor in a conversation when a patient feels less self-assured about his/her decision making skills (Hamann et al. 2011).

The patients in our sample wanted to be involved in the decision process about their treatment options, in line with previous studies (Cuevas et al. 2014; Klingaman et al. 2015). When in the end the final decision has to be made, we see a pattern emerging regarding differentiation by age. It appears that being older predicted stronger preferences for deciding together or ‘letting the doctor decide’. These results were also found by Cuevas et al. (2014). This could be explained by the fact that a few decades ago, a more passive decision making style was commonly used, so older patients are used to this. However, our study was not designed to study this hypothesis. It might be interested to investigate this further in future research.

If patients were (theoretically) forced to choose who made the final decision, half of them would prefer to make it on their own and the other half would prefer the doctor to decide. This was also found by, Park et al. (2014) who showed that more than half of patients preferred the doctor to decide. Therefore, it seems that patients in our sample do want to be involved *in the process*, but not so much in the *actual decision* made at the end. Note that none of the clinicians in our sample discussed explicitly, on a meta-level, the patient’s preferences with regard to making health decisions in general.

### Strengths and limitations

The strength of this study is its focus on one specific psychiatric disorder and only on pharmacotherapy decisions. Furthermore, this is the first study to explore the occurrence, needs and expectations of SDM in this number of only bipolar patients. The few studies which also included bipolar-only samples were aimed at researching the quality of patient-clinician interactions and the influence of SDM on clinical outcomes (Ilgen et al. 2009; Sylvia et al. 2013; Sajatovic et al. 2005).

The results of this study should be read with some limitations in mind. We used methods in order to find a quick overview of patient experiences and opinions of SDM. Further qualitative analyses of patient-clinician interactions are necessary to deepen our understanding of how patients and clinicians take a position in decisions.

It is important to note that the OPTION scale only scores the clinician’s behaviour and does not directly score the interaction between patient and clinician. Imagining the most extreme situation, in which an ‘empowered’ patient enters the consultation room and spontaneously states his or her opinion and concerns about the treatment options, the clinician would score zero on several scoring points, resulting in the overall SDM score being lower. Future research methods should focus primarily on the interaction between patients and clinicians.

Finally, it should be mentioned that no data was available about the socio-cultural-economic background of the patients. Thereby the influences of these variables are not researched in this study.

### Conclusion and recommendations

The patients with bipolar disorder in our sample want to be involved in pharmacotherapy decisions, especially younger patients. We advise clinicians to tailor SDM and actively explore the patient’s preferences with regard to information, the style of the decision process, and the final call in the decision.

In accordance to Fisher et al. (2016), Goossensen et al. (2007) and Etwistle et al. (2006), our data suggest that there are implicit and explicit aspects of patient involvement in SDM, and the two aspects are uncorrelated. Research and policy on SDM so far seem to focus predominantly on the objective part. The interpersonal part may not have been addressed sufficiently in the literature. Clinicians and patients might deduce each other’s treatment options and wishes in a more intuitive manner, based for example on their previous treatment history or non-verbal signals. Future (qualitative) research could deepen our understanding of the interactions that take place between clinicians and patients in decision making and should develop new measuring scales to make this implicit part observable.

Our quantitative data suggest that age, self-efficacy and duration of the consultation may be variables to consider in further research.

Finally, modern health care is shifting from doctor-centred to patient-centred care. SDM, our dominant paradigm in decision making, is more than the exchange of information and values. There are indications that there is an interpersonal aspect to SDM in the consultation room which deserves the attention of researchers, clinical training providers and policy makers.

## Abbreviation

SDM: shared decision making
